# Novel Micro-Nano Optoelectronic Biosensor for Label-Free Real-Time Biofilm Monitoring

**DOI:** 10.3390/bios11100361

**Published:** 2021-09-29

**Authors:** Giuseppe Brunetti, Donato Conteduca, Mario Nicola Armenise, Caterina Ciminelli

**Affiliations:** 1 Optoelectronics Laboratory, Department of Electrical and Information Engineering, Polytechnic University of Bari, 70125 Bari, Italy; giuseppe.brunetti@poliba.it (G.B.); donato.conteduca@york.ac.uk (D.C.); marionicola.armenise@poliba.it (M.N.A.); 2 Photonics Group, Department of Physics, University of York, Heslington, York YO10 5DD, UK

**Keywords:** bacteria biofilm, optoelectronic device, antimicrobial resistance, biosensing

## Abstract

According to the World Health Organization forecasts, AntiMicrobial Resistance (*AMR*) is expected to become one of the leading causes of death worldwide in the following decades. The rising danger of *AMR* is caused by the overuse of antibiotics, which are becoming ineffective against many pathogens, particularly in the presence of bacterial biofilms. In this context, non-destructive label-free techniques for the real-time study of the biofilm generation and maturation, together with the analysis of the efficiency of antibiotics, are in high demand. Here, we propose the design of a novel optoelectronic device based on a dual array of interdigitated micro- and nanoelectrodes in parallel, aiming at monitoring the bacterial biofilm evolution by using optical and electrical measurements. The optical response given by the nanostructure, based on the Guided Mode Resonance effect with a *Q*-factor of about 400 and normalized resonance amplitude of about 0.8, allows high spatial resolution for the analysis of the interaction between planktonic bacteria distributed in small colonies and their role in the biofilm generation, calculating a resonance wavelength shift variation of 0.9 nm in the presence of bacteria on the surface, while the electrical response with both micro- and nanoelectrodes is necessary for the study of the metabolic state of the bacteria to reveal the efficacy of antibiotics for the destruction of the biofilm, measuring a current change of 330 nA when a 15 µm thick biofilm is destroyed with respect to the absence of biofilm.

## 1. Introduction

Bacterial infections represent one of the leading causes of death in developing nations [1]. The infections are caused by food poisoning, which is often related to water contamination or improper food preparation [2]. Furthermore, the large overuse and/or misuse of antibiotics is causing a rapid growth in AntiMicrobial Resistance (*AMR*) worldwide [3]. *AMR* is developed when the bacteria adapt to and resist antibiotics treatments, which become ineffective to counteract a bacterial infection that can grow and spread in a large community through direct contact, food, or the environment [4,5]. As a result of the lack of powerful antibiotics, many bacterial infections, such as pneumonia, tuberculosis, and gonorrhoea, are becoming more difficult to eradicate with a consequent higher mortality rate [6]. According to [7], the cost of *AMR* on public health is up to 100 trillion USD, and *AMR* is expected to become the leading cause of death worldwide, with over 10 million annually predicted by 2050. These consequences highlight that *AMR* is a widespread social problem that cannot be underestimated or neglected anymore due to the large and rising number of people potentially affected.

Many bacterial infections are caused by the non-eradication of bacterial biofilm, which can be several times more resistant to antibiotics compared to planktonic bacteria [8,9]. This behavior is strictly correlated to the intrinsic nature of the biofilm, which consists of densely packed microbial cells that can grow and surround themselves with a self-produced Extracellular Matrix (*ECM*). The *ECM* is composed of proteins, polysaccharides, and nucleic acids that protect the bacterial biofilm from the environment, so making it very resistant to external agents, such as antibiotics [10]. Moreover, a biofilm may include different bacteria, and this makes the dissolution of biofilms more challenging [11]. In fact, it has been demonstrated that even if a biofilm is treated by an antibiotic that is efficient for a specific planktonic bacterium or small communities of bacteria with a concentration much higher than Minimum Biofilm Inhibitory Concentration (*MIC*), which represents the lowest concentration of drug to prevent the bacteria growth, the biofilm structure can be completely unaltered, showing a continuous growth process also after the treatment [12].

## 2. Techniques for the Bacteria Detection and Analysis

To date, the most widely used diagnostics to detect the presence of bacteria and analyze their evolution under several antibiotics’ treatment is the plate-count method, which is based on the growth of bacteria on an agar plate [13,14]. However, this technique is time consuming (24–72 h), because it requires many cell-division cycles as well as expert users for the sample preparation and final analysis. The large time delay is the most significant bottleneck of such a technique because several infections, such as sepsis, require an immediate measure, also to avoid the formation of a biofilm [15]. The antibiotics are commonly administered in the clinicians’ experience with a not-negligible failure rate, so possibly leading to an outbreak of the resistance rather than by carrying out an accurate diagnosis. Thus, novel diagnostic techniques that can rapidly detect and identify bacteria and confirm the presence of a biofilm, ideally within 30 min, are needed. Furthermore, these techniques should also enable study of the efficiency of antibiotics with a real-time analysis during the treatment in order to define the most powerful antibiotic, the best concentration, and the administration time for each infection [16].

During the last few years, several approaches have been investigated, mainly with the use of optical techniques, such as Raman Spectroscopy or fluorescence, due to their high resolution and real-time detection of individual bacteria [17,18,19]. However, these methods are not label-free, becoming inefficient in the presence of bacteria mutations, and allow investigating only a small area. Integrated optical devices have also been used for single bacteria analysis with label-free techniques [20]. In particular, resonant cavities are able to trap and identify single bacteria through the changes of the resonance response [21,22]. Emerging studies with a label-free optical-based approach have demonstrated real-time monitoring of cell attachment and the development of bacteria on the sensor surface [23]. Optical devices have been used to investigate the antimicrobial susceptibility with in vitro studies by adopting antibiotics with concentrations compliant with standard health protocols [23]. However, the simultaneous detection of several bacteria in the whole biofilm volume is still challenging because of the mismatch between the biofilm thickness and evanescent field penetration depth. In fact, the evanescent field of resonant cavities typically extends for few hundreds of nanometers, thus making impossible the detection of multiple bacteria organized in a three-dimensional configuration as a biofilm that can reach a thickness of several microns in the presence of macro-colonies in the maturation phase.

The mechanical trapping of bacteria has also been obtained by using microfluidic devices [24,25], allowing the bacteria localization in specific areas to accurately detect and analyze them with an atomic force microscope (*AFM*) [26]. The *AFM* technique guarantees a very high resolution, also providing relevant information about the metabolic state of bacteria and if they are live/dead, for example by observing a different motility with a change of amplitude and noise in the electrical signal [26]. However, the mechanical trapping has a trapping time not long enough to investigate the metabolic activity of bacteria and their interactions in large communities, so making also difficult their differentiation in the biofilm.

The aforementioned critical issues have been partly mitigated by Electrochemical Impedance Spectroscopy (*EIS*) [27,28,29,30,31]. In particular, device configurations based on interdigitated microelectrodes have been exploited for the analysis of the metabolic activity of bacteria and their differentiation [27]. This approach is characterized by a great penetration of the electric field in the biofilm, in contrast to the integrated optical devices where the distribution of the evanescent field of the optical resonant mode within the biofilm is limited [32], as demonstrated in the next sections. Therefore, the electrical behavior guarantees the capability of studying the biofilm evolution and the action of the antibiotics.

However, the lowest detection limit of *EIS* is around 10 CFU/mL [31], which means no high resolution down to single cells. Moreover, the detection time is longer compared to other trapping approaches, such as optical and mechanical ones, because, in case of low initial concentration, the growth of the cells is required before achieving a detectable change of impedance, which usually takes a few hours [33].

The resolution can be improved by different configurations of electrochemical biosensors, such as interdigitated nanoelectrodes, which compared to the bulk configuration of single electrodes allow the enhancement of the electric field in the device and then, a stronger interaction between the field and the bacteria [34]. However, if the resolution improvement is achieved at the expense of a shorter penetration length of the electric field, the analysis of the bacteria metabolism in a biofilm would be impossible.

From this brief overview on the techniques for the detection and analysis of bacteria, it is clear that a single interrogation technique is not sufficient for a full assessment of the antibiotic susceptibility on planktonic bacteria and, in particular, on bacterial biofilms. Multiple approaches should be used in parallel, leading to a multiparameter approach [35,36].

Here, we propose the design of a novel optoelectronic device based on an on-chip dual array of interdigitated micro- and nanoelectrodes that combines together optical and electrical techniques to monitor the growth of a biofilm and to analyze the effect of antibiotics on the bacteria. The optical approach allows the detection of few bacteria with a high spatial resolution to understand their interaction and which biological events are involved during the initial phase of the biofilm formation. The electrical approach allows the simultaneous study of the evolutionary phases of the bacterial biofilm and, by analyzing the impedance changes, the evaluation of the biofilm growth and maturation, and the efficacy of antibiotics for the disruption of the biofilm, which are useful in AMR studies.

## 3. Dual Array of Interdigitated Electrodes: Architecture and Operation

**Figure 1:**
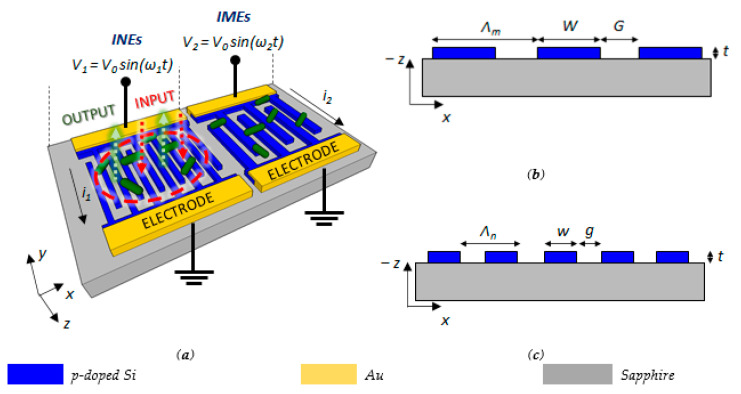
(**a**) Configuration of the dual array of interdigitated micro- and nanoelectrodes in SOS technology for the electrical detection of bacteria (in green) growth and metabolism. The section with nanoelectrodes represents an optical sub-wavelength grating resonating at a specific wavelength when top illuminated, so enabling a simultaneous optical and electrical detection of bacteria deposition; (**b**) Interdigitated Micro Electrodes (*IMEs*), where *W* is the width of the silicon layer (*W* >> *w*), *G* is the gap between electrodes (*G* >> *g*); and *Λ_m_* is the *IMEs* period; (**c**) Interdigitated Nano Electrodes (*INEs*), where *w* is the width of the silicon layer, *g* is the gap between electrodes; and *Λ_n_* is the *INEs* period. The output signal of the Guided Mode Resonance (GMR) structure consists of the reflected optical signal (green arrows) by illuminating the optical section with *TE*-polarized light (red arrows).

The dual array is formed by an Interdigitated Micro Electrodes (*IMEs*) section and an Interdigitated Nano Electrodes (*INEs*) section, which are both realized in Silicon-On-Sapphire (*SOS*) technology and fed by an *AC* voltage (see Figure 1a). In order to perform both optical and electrical measurements to study the properties of planktonic bacteria or a bacterial biofilm in the whole sensor area, the two sections are arranged adjacent, spaced from each other only by a few microns.

P-doping has been assumed for silicon, with an exponential decay of the electrical conductivity from the surface in depth with a drop more than three orders of magnitude in few tens of nanometers, as described in Section 4.1, reaching very low values of resistivity at the surface, and also strongly reducing the optical losses correlated to the dopant concentration [37]. The *INEs* section can be assumed as a top-illuminated subwavelength grating, whose cross-section is sketched in Figure 1, that supports the Guided Mode Resonance (*GMR)* effect [38]. It is correlated to the quasi-guided modes or leaky modes of the structure, as shown by the grating in Figure 1a. The grating acts as a waveguiding layer in the *x-y* plane, where the input light excites quasi-guided or leaky modes that coherently scatter at each interface of the grating. Furthermore, leaky modes scatter power downwards, along the vertical direction perpendicular to the grating (*z*-axis).

By properly engineering the grating features, such as the period, the refractive indices, and the angle of incidence, the interference between the transmitted light and the downward wave scattered by the leaky mode could generate reflected light along the negative direction of the *z*-axis with maximum amplitude at resonance [39,40].

The structure exploiting the *GMR* effect has been designed to obtain a resonance condition for *λ* > 800 nm, where the absorption losses of silicon decrease [41], with the aim of achieving a higher extinction ratio and higher *Q*-factor, together with a strong energy confinement close to the surface to enhance the light interaction with the bacteria. The strong refractive index contrast between silicon and the surrounding medium allows a high confinement of the electromagnetic field at the sensor surface, enabling the use of the *INEs* section for hyperspectral imaging technique, as described in detail in [42], which allows the refractive index imaging, thus localizing objects on the grating by detecting the spatial resonance distribution. An inverted microscope configuration can be used to characterize the sensor, with the light source illuminating from the top and the reflected signal collected from the same side of the setup by a conventional CMOS camera.

In order to work at the bacteria scale, we assume as a best compromise in terms of resolution and the large field of view an optical setup with an area of 1 mm^2^ and a spatial resolution down to few microns. This allows having a large area but still exploiting the advantages of near-field optics, in particular in terms of strong resolution. This approach has been experimentally validated in the literature with simple and feasible systems [42]. This behavior is also useful to obtain additional information about the first stage of the bacteria infection, when the bacteria cells enrich before they start to interact with each other to form the biofilm. During the biofilm formation, the cells produce extracellular polymeric substances (*EPS*), creating the surrounding matrix to protect the bacteria. The main *EPS* components are polysaccharides, proteins, lipids, and *DNA* with dimensions much smaller than the bacterial cells [43]. The real-time detection of the biofilm formation with label-free techniques is very challenging, mainly because the matrix generation begins when the first layers of bacteria close to the sensor surface are packed and arranged in large communities. Under this condition, the monitoring of the chemical and biological processes in these communities is difficult because the propagation length of the evanescent field, of the order of few hundreds of nanometers, corresponds only to the first layer of bacteria. Therefore, the optical approach is efficient for analyzing the first stages of the biofilm formation when bacteria begin to form colonies, while a different approach should be investigated to clearly define the presence of a biofilm with its extracellular matrix. To meet this latter requirement, the *INEs* section has been designed to allow simultaneous optical and electrical measurements. In fact, several pairs of nanoelectrodes can be realized in an interdigitated configuration, by connecting in turn the doped silicon structures to two different metal electrodes, as shown in Figure 1c, so forming several capacitors at the nanoscale. This system of interdigitated electrodes can allow easy monitoring of the large change of the *PH* and the electrical properties of the solution induced by the secretion of proteins, *DNA,* and other *EPS* components when the bacteria colonies start to create the extracellular matrix [44]. In particular, the capacitance of the system changes when the bacteria start to grow because of the strong interaction between the electric field and the bacteria. Low-frequency values are usually used to detect changes of the system capacitance, because under this condition, the bacterial membrane behaves as a barrier to prevent the penetration of the electric field into the cytoplasm of the bacteria, which usually has a much higher conductivity, in order to make more evident any change of the impedance in proximity of the electrodes. The *INE* section supporting the *GMR* effect guarantees the confinement of the electric field close to its top surface [34], where the bacteria grow and the chemical processes happen, with an improved sensitivity compared to other electrical approaches with different systems and configurations.

Therefore, the *INEs* provide high spatial resolution for imaging few bacteria through an optical approach, to investigate the bacteria interaction to form colonies, and an electrical approach to analyze the biofilm evolution, due to the presence of *EPS* components that change the electrical properties of the solution, and, therefore, interfere with the electric field distribution. This interaction induces a change of the capacitance *C_i_* of the nanocapacitor formed by each pair of nanoelectrodes, which depends on the electrical properties of the surrounding medium and the electrode geometry [44]. The net capacitance of the system, *C_tot_*, is given by the sum of the single capacitances *C_i_* in parallel combination.

A voltage of few millivolts and low frequency have been assumed, to have a low power interacting with bacteria with consequent strongly reduced risks, such as bacteria membrane collapses or changes of their metabolic state. An *AC* signal at low frequency, i.e., *f*_1_ ≈100 Hz, is necessary to detect any change of the system capacitance [45]. The net impedance of the system is given by *Z* = 1/j*ωC_tot_* = 1/j*ωNC_i_*, where *N* is the number of nanocapacitors in the interdigitated configuration. This corresponds to a maximum value of current *i_max_* = *V*_0_/|*Z*| = *ωV*_0_*NC_i_*, which can be increased with the number of pairs of electrodes, leading to the increase in the Signal-to-Noise Ratio (*SNR*) and the sensitivity.

Since a higher sensitivity also corresponds to a faster saturation of the impedance change, due to the strong confinement of the electric field, which is not affected by the impedance changes in the upper layers of the biofilm, the main limitation of the *INEs* is given by the narrow dynamic range [46]. To monitor the biofilm upper layers, a great penetration depth of the electric field is needed, with a resulting large dynamic range and a lower sensitivity. For this reason, the sensor also includes an *IMEs* section placed next to the *INEs* structure (see Figure 1a). Since the penetration depth *L* of the electric field is proportional to the gap *G* and to the width *W* of the electrodes (*L*–*W*), features at the microscale lead to a large penetration depth and dynamic range [47]. In particular, by assuming *W* > 10 μm, any change of the metabolic state of the biofilm can be monitored as an impedance change. To improve the *SNR*, an *IMEs* driving voltage frequency *f*_2_ larger than 1 kHz can be used, where the biofilm shows resistive behavior. According to the above-mentioned net impedance equation, an increase in the electrodes’ driving voltage leads to a decrease in the impedance *Z*, allowing improvement in the accuracy of the detected changes in the biofilm with respect to the *INEs* section. 

Although *INEs*- and *IMEs*-based biosensors have been already widely investigated, see as examples [48,49], we note that the main novelty of the proposed optoelectronic device is the combination of both optical and electrical approaches to perform on the same platform and at the same time the efficient monitoring of the bacteria growth and the analysis of the resulting biofilm under antibiotics treatment. According to the above, it can be said that the high sensitivity is provided by *INEs* through a strong confinement of the electric field close to the top silicon surface, while the *IMEs* allow analyzing the growth and the maturation of the biofilm and studying its full or partial disruption induced by the antibiotic treatment for a complete, accurate, and real-time monitoring of the biofilm properties and analysis of *AMR* to specific antibiotics. The driving voltages *V*_1_ and *V*_2_ for *INEs* and *IMEs*, respectively, can be the same to simplify the setup of the system, while an *IMEs* driving voltage frequency *f*_2_ larger than the *INEs* one is preferred to improve the SNR.

The system configuration could be realized following typical fabrication processes. For example, a lithographic process followed by reactive-ion etching can be used to define both the *INEs* and *IMEs* structures, while photolithography followed by metal evaporation and lift-off can be applied for the metal pads (Figure 1).

## 4. Design of the Dual Array of Interdigitated Electrodes

### 4.1. Design of the INEs for Optical and Electrical Measurements

**Figure 2:**
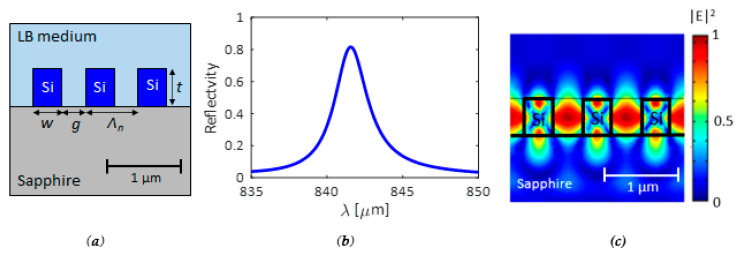
(**a**) Configuration of the *GMR* structure (*LB*: Lysogeny Broth); (**b**) Reflection spectrum; (**c**) Energy confinement at the resonance wavelength λ ≈ 841.5 nm.

The optical section, as already mentioned, consists of a *GMR* structure with a subwavelength grating in SOS technology. A doped silicon was assumed for doing electrical measurements, with a dopant concentration *N_D_* = 10^21^ cm^−3^ at the surface and an exponential drop-off to *N_D_* = 10^18^ cm^−3^ within 20 nm, which can be achieved by thermal diffusion doping [37,50]. This doping profile allows minimizing the optical losses, without any worsening of electrical performance. The geometrical features of the silicon subwavelength grating have been designed to enhance both electrical and optical performance. A resonance condition around *λ* = *λ_res_* ≈ 850 nm is required to minimize optical losses due to the material absorption typical for silicon at lower wavelengths. To fulfill all the requirements, including fabrication tolerances, we have determined a thickness *t* = 270 nm, a period *Λ_n_* = 440 nm, and a fill factor *FF* = 0.5, corresponding to *w = g* = 220 nm (see Figure 2a). Furthermore, since the grating strength, and then the *Q*-factor, increases with the number of periods, thousands of periods have been assumed, without affecting the ability to detect the bacteria with a resolution of a few microns.

The reflection spectrum of the grating has been calculated by the 3D Finite Element Method (*FEM*), assuming top out-of-plane excitation with *TE*-polarized and plane-wave collimated light (the electric field is oriented perpendicularly to the grating period direction).

Reflection spectrum and mode distribution at the resonance frequency are reported in Figure 2b,c, respectively.

The Lysogeny Broth (*LB)* (*n_LB_* = 1.333 + 5 × 10^−7^ i) has been assumed as the surrounding medium, which is a typical medium for bacteria, and the substrate is sapphire with *n_sub_* = 1.732. A resonance condition at *λ_res_* ≈ 841.5 nm has been calculated. At *λ_res,_* with the aforementioned doping profile, a refractive index of silicon *n_Si_* = 2.76 + 0.06 i at the surface results, which increases up to *n_Si_* = 3.648 + 4 × 10^−3^ i at 20 nm far from the surface. The resonance shows an amplitude higher than 0.8, which is normalized with respect to the input power, and a Full Width at Half Maximum (*FWHM*) = 2.12 nm, which corresponds to *Q* ≈ 400. The proposed configuration represents the best compromise to achieve a high *Q*-factor, large modal confinement, and high resonance amplitude, which is useful for hyperspectral imaging and sensing.

**Figure 3:**
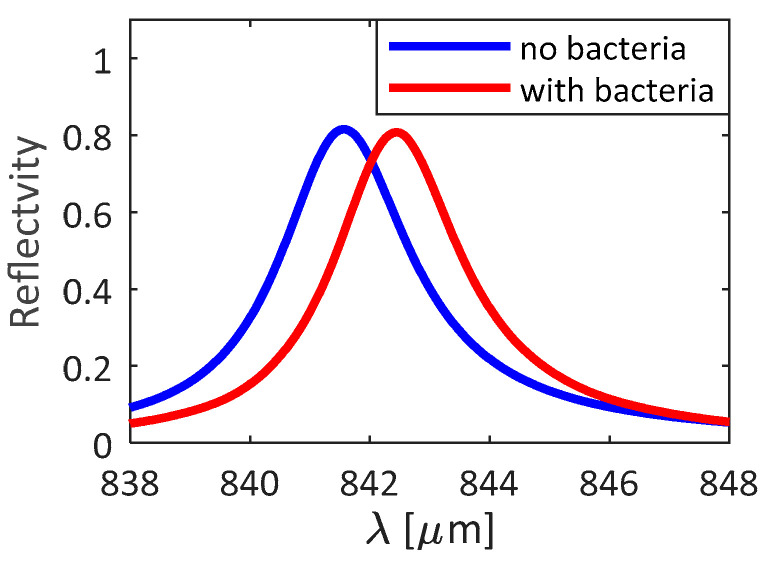
Reflection spectra for the case without bacteria (blue curve) and with bacteria (red curve) on the *GMR* structure. Spectra calculated by 3D FEM approach.

The operation of the *GMR* structure in the presence of bacteria has been simulated by assuming a uniform and homogeneous layer of bacteria with a thickness *t_layer_* of the order of hundreds of nm and refractive index *n_bac_*. For example, the *Escherichia coli* bacterium was considered, with a diameter *t_bac_* of 500 nm, a length *l_bac_* of 2 µm, and a refractive index *n_bac_* = 1.388 [51]. Since the length of the bacteria *l_bac_* is larger than the gap size *g*, the penetration of bacteria in the grooves is not allowed. Therefore, the grooves have been assumed to be filled by *LB* medium. The comparison between the 1D silicon *GMR* surrounded by *LB* only and the same structure with the presence of bacteria in solution is shown in Figure 3. A resonance shift *Δλ_res_* of about 0.9 nm in the case of the bacteria layer on the *GMR* structure, with a reflection change of about 0.02, was evaluated. This behavior confirms a high resolution in detecting the presence of the bacteria, even when the surface is not fully covered.

Since the evanescent field of the optical mode extends and interacts with the biofilm for a few hundred nanometers (<<*t_bac_* = 500 nm), beyond the first layer of bacteria, the optical response results are insensitive to an increase in the layer thickness, as confirmed by FEM simulations where the behavior of the reflected signal is the same for *t_layer_* ≥ *t_bac_* with negligible resonance shifts. However, during the biofilm formation, a clearer resonance shift is also expected because of the release of small particles and molecules, possibly in the grooves, which would further affect the effective index. As an example, in the extreme case of filling the grooves with biomolecules secreted by the *Escherichia coli* bacteria (for which we assume the same refractive as for the bacteria, *n_bac_* = 1.388), 3D *FEM* simulations confirm a maximum resonance shift up to 7.5 nm. Therefore, in the presence of a biofilm, a final value of the resonance shift in the range 0.9 nm < Δ*λ_res_* < 7.5 nm is expected.

When investigating the resonance shift in an array of several pixels, the resonance map for each pixel can give information about the position of bacteria and the size of colonies.

**Figure 4:**
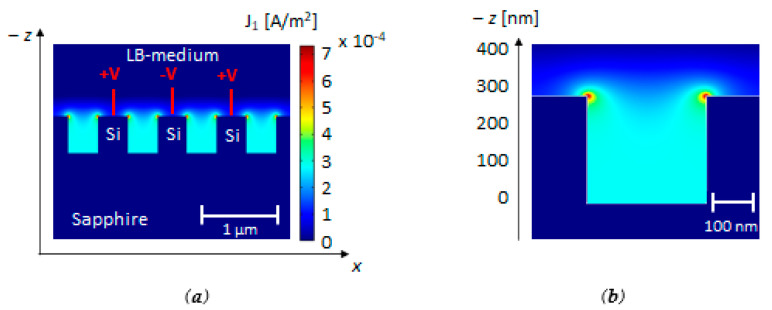
(**a**) Current density *J*_1_ [A/m^2^] distribution along the *x*-axis in the middle of the *INEs* structure with *f*_1_ = 100 Hz, *V*_0_ = 10 mV, *Λ_n_* = 440 nm, and *FF* = 0.5; (**b**) Focus at the surface of the electrodes with the energy confinement in few hundreds of nanometers. Plots calculated by 2D FEM approach.

However, due to the short penetration length of the evanescent field in the surrounding medium, this approach cannot provide a very accurate analysis about the formation of a biofilm and its maturation. This limitation justifies the choice of a more complex biosensing platform by combining optical measurements with electrical ones. Hence, the same interdigitated silicon nanoelectrodes have also been used for electrical measurements, as shown in Figure 1a, where an applied voltage *V*_0_ = 10 mV enables the current flow *i*_1_. In order to not interfere with the optical response, the metal pads of this structure are far enough from the region used for optical measurements. An electrode length of about 1 mm fulfills this requirement, also avoiding any power absorption of the medal pads, with a consequent reduction of the interacting optical power. The main goal of the *INE* structure is detecting the presence of bacteria and the first stage of bacterial biofilm formation. The maximum sensitivity can be reached by evaluating the changes of the system capacitance, where the capacitance is given by *C* = *εA*/*d*, with *ε* being the relative permittivity of the surrounding medium, *A* being the area of the electrodes, and *d* being the distance between the silicon surface and the charged particles released by the bacteria [52]. Electrical measurements require strong confinement of the electric field at the surface of silicon, which is achievable with a typical frequency *f*_1_ ≈ 100 Hz. The relative permittivity of *LB* medium at *f*_1_ = 100 Hz has been assumed to be equal to *ε* = 60, and the electrical conductivity *σ_LB_* = 0.754 S/m. As the first step, the electrical behavior of the *INEs* has been evaluated by 2D *FEM* simulations without the presence of bacteria to observe the electric field distribution in the proximity of the electrodes. The grating length is much larger than the grating period and the electrodes features; therefore, we have assumed a 2D simulation as an optimal approximation of the real case of study. Figure 4 shows the distribution of the current density *J*_1_ [A/m^2^] without bacteria. As expected, the energy decreases as a function of the distance along the *z*-axis from the electrodes and increases with a peak of the energy density (≈7 × 10^−4^ A/m^2^) at the silicon ridges. This behavior confirms the suitability of the electrical measurements to monitor the biofilm at the silicon surface.

The total capacitance of the *INE* structure is directly proportional to the number of pairs of electrodes. A number of couples *N* = 2000 and a length of electrodes of the order of mm have been assumed in the model, which corresponds to a total width of the system of *N*·*Λ* = 2000 × 0.44 μm = 880 μm. The footprint of the order of mm^2^ (=880 μm × ≈1 mm) also guarantees a large area of the *INEs* section optimizing the optical reflection and obtaining more information for large bacterial colonies. The capacitance of a system with interdigitated electrodes is [53]:

(1) $$C=\frac{N\cdot Q}{{2V}_{0}}=N\cdot \frac{2}{V_{0}{}^{2}}E=N\cdot \frac{2}{V_{0}{}^{2}}\int_{\Omega }W_{e}d\Omega$$

where Ω is the surrounding area of the electrodes close to their surface [m^2^], *E* is the energy for each pair of electrodes [J], and *W_e_* is the electric energy density [J/m^2^]. Assuming *V*_0_ = 10 mV and *f*_1_ = 100 Hz, the energy *E* is equal to 31 fJ, which corresponds to a total system capacitance *C* = 1.24 μF. In the low-frequency regime (*f*_1_ = 100 Hz), the system behavior is capacitive, with a corresponding impedance *Z_c_ =* 1*/jωC*, decreasing as *C* increases. *N* = 2000 corresponds to *Z_c_* = 1.2 kΩ. The maximum value of the current *i*_1*,max*_ is given by [54]:

(2) $$i_{1,\max\;}=\max\left(C\frac{\partial V}{\partial t}\right)=\max\left(C\frac{\partial {(V}_{0}\sin(\omega t))}{\partial t}\right){=2\pi f}_{1}{\mathrm{CV}}_{0}=7.79\;\mu A.$$

To simulate the biofilm formation and maturation in the *LB*, the well-established Maxwell Mixture Theory (*MMT*) [55] has been used to define the electric properties of the biofilm. In particular, the *MMT* method assumes the biofilm as a compound of uniformly distributed spherical objects as the bacterial cells, which are covered by a shell to mimic the external membranes, forming the so-called Extracellular Matrix (*ECM*) [46]. With these assumptions, the dielectric permittivity and electrical conductivity of the biofilm can be theoretically estimated. In particular, the relative permittivity of the region with the biofilm ε*_biofilm_(ω) is given by [55]:

(3) $${\varepsilon *}_{\mathrm{biofilm}}{(\omega )=\varepsilon *}_{\mathrm{ECM}}\left(\omega \right)\frac{{2(1\;-\;\varphi )\varepsilon *}_{\mathrm{med}}{\;+\;(1\;+\;2\varphi )\varepsilon *}_{\mathrm{eq}}\left(\omega \right)}{{(2\;+\;\varphi )\varepsilon *}_{\mathrm{med}}{\;+\;(1\;-\;\varphi )\varepsilon *}_{\mathrm{eq}}\left(\omega \right)}$$

where *φ* is the fractional volume of the bacterial cells in the *ECM* that has been assumed equal to 30% following a conservative approach [45], 𝜀*_MED_ is the complex permittivity of the solution where the bacteria are immersed, and ε*_eq_(*ω*) is the equivalent complex dielectric constant of the bacteria, expressed as [55]:

(4) $${\varepsilon *}_{\mathrm{eq}}{(\omega )\;=\;\varepsilon *}_{\mathrm{mem}}\left(\omega \right)\frac{{2(1\;-\;\theta )\varepsilon *}_{\mathrm{mem}}{\;+\;(1\;+\;2\theta )\varepsilon *}_{\mathrm{cyt}}\left(\omega \right)}{{(2\;+\;\theta )\varepsilon *}_{\mathrm{mem}}{\;+\;(1\;-\;\theta )\varepsilon *}_{\mathrm{cyt}}\left(\omega \right)}$$

with the complex permittivity of the ECM ε*_ECM_(*ω*) = *ε_r_ECM_* + *σ_ECM_/(jε_0_ω)*, the complex permittivity of the bacterial cell membrane ε*_MEM_(*ω*) = *ε_r_MEM_* + *σ_MEM_/(jε_0_ω)*, and the permittivity of the bacterial cytoplasm ε*_CYT_(*ω*) = *ε_r_CYT_* + *σ_CYT_/(jε_0_ω)*. Moreover, *θ = (R/(R + d))*, with *R* and *d* being the radius of the bacteria and the thickness of the external membrane, respectively, and the parameters *ε_0_* and *ω* being the dielectric permittivity in vacuum and the angular frequency of the applied signal, respectively. The conductivity of the biofilm is calculated as *ε_biofilm_* = *ε_r_biofilm_ + σ_biofilm_/(jε_0_ω)*. According to the *MMT* theory, a negligible change would be obtained with a model that assumes bacteria with an ellipsoidal shape instead of a spherical one. The parameters values used in the numerical model to implement the *MMT* are reported in Table 1. The electrical properties are derived by experimental measurements reported in the literature [46,56]

An initial value of the capacitance *C*_0_ = 1.24 μF has been simulated by assuming only *LB* medium above the nanoelectrodes. As already assumed in the optical analysis, the presence of a biofilm layer with a thickness of 1 μm, above the nanoelectrodes, was considered. Under this condition, the capacitance becomes *C’* = 1.41 μF, the capacitance relative change is Δ*C/C*_0_ = (*C’* − *C*_0_*)/C*_0_~14%, and the maximum value of current is equal to *i*_1*,max*_ = 8.85 μA. This performance confirms the high sensitivity of the nanoelectrodes because a change of the current values of about 14% is obtained when a single layer of bacteria is placed on top of the nanoelectrodes (thickness = 1 µm). This behavior is strictly correlated to the strong confinement of the electric field at the surface of the electrodes and represents a significant advantage with respect to optical measurements in terms of sensitivity to the biofilm formation and maturation. However, the electrical measurement takes into account only an average change of the surrounding medium with a spatial resolution > 1 µm, while the optical approach provides the spatial distribution of bacteria along the grating with a much higher resolution, of the order of hundreds of nm, so demonstrating the strong complementarity of the methods. Since the strong electric field confinement of the *INE* structure causes the saturation of the impedance value, even with a single layer of bacteria in the biofilm, negligible changes of the impedance for a thicker layer of biofilm were confirmed by *FEM* simulations. This restriction justifies the combination of the *INE* and *IME* structures in order to also detect a thicker biofilm for the study of the maturation phase and for the analysis of a possible biofilm disruption by using antibiotics whose results are challenging only with the *INE* structure.

### 4.2. Design of the IMEs for the Detection of Biofilm Maturation or Disruption

**Figure 5:**
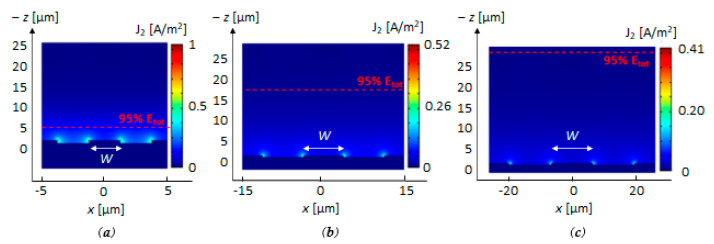
Current density *J*_2_ [A/m^2^] distribution in the *IME* structures with a value of width *W = G* of 5 μm (**a**), 15 μm (**b**), and 25 μm (**c**). The current values have been normalized to the maximum value of current calculated for *W* = 5 μm. The dotted red line represents the distance from the electrodes, where 95% of the total energy is confined. Plots calculated by 2D FEM approach.

The same thickness and doping distribution of the *INEs* described in Section 4.1 were assumed for the configuration of the *IMEs* to realize the proposed array with a single manufacturing process. As above introduced, the *IMEs* function is providing an accurate analysis of the biofilm maturation and its possible disruption through an interaction of the electric field distribution with the upper layers of the bacterial biofilm. A design different from *INEs*, in terms of width *W*, gap *G* between the electrodes, and period *Λ_m_* (see Figure 1c) is required. The electric field distribution for different values of width *W* is in Figure 5, for different values of W (*W =* 5 μm, 10 μm, and 15 μm with *W = G*). The numerical results confirm that a larger value of *W* and *G* allows confining the electric field farther away from the electrodes, as required for the *IME* structure, at the expense of a decrease in the current density *J*_2_ [A/m^2^] due to a worsening of the related capacitance value.

**Figure 6:**
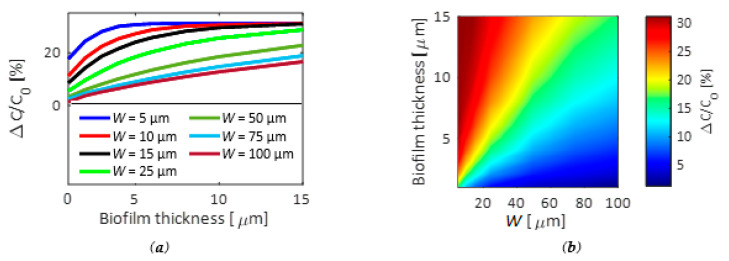
(**a**) Change of the capacitance Δ*C/C*_0_ *=* (*C’* − *C*_0_*)/C*_0_ in the *IME* structure for different values of thickness of the bacterial biofilm as a function of the electrode width (*W = G*); (**b**) Change of the capacitance Δ*C/C*_0_ as a function of the electrode width calculated for different values of the biofilm thickness.

The performance of the *IME* configuration without the bacterial biofilm was defined by *FEM* simulations for different values of the width, assuming a number of electrodes *N* = 100, aiming at preserving the device compactness. Figure 6 shows the change of the capacitance for different values of *W* (assuming *G = W*) with respect to the capacitance *C*_0_ calculated when the *IMEs* are not covered by the biofilm layer.

The same model based on the *MMT* has been used to define the electrical performance for *IMEs*, as already proposed for the nanoelectrodes. The presence of multiple layers of bacteria has been considered for the biofilm, with each layer of bacteria assumed with a thickness of 1 μm.

As expected, the capacitance changes quickly for small gap values due to the stronger energy confinement, but this also corresponds to a more evident nonlinear behavior for a thickness of the biofilm of few microns (see Figure 6a), which makes the rigorous analysis of the biofilm behavior challenging, even with several layers of bacteria.

For example, a value of *G = W* = 5 μm presents a nonlinear behavior up to 5 μm, where the capacitance reaches its saturation value. For a biofilm thickness larger than 5 μm, the changes of impedance are negligible, making impossible the analysis of the upper biofilm layers. On the contrary, a larger value of *G* provides a more evident linear behavior of the capacitance change with respect to the thickness of the biofilm at the expense of less sensitivity. In fact, for *G = W =* 100 μm, a linear behavior of the capacitance has been observed for a thickness of the biofilm of at least 15 μm. However, an impedance change of about 10% has been calculated for a thickness of the biofilm of 10 μm, while the impedance variation goes down to 5% with a thickness of 5 μm, which is six times lower than the performance obtained with *G* = 5 μm for the same biofilm thickness.

Figure 6b confirms the nonlinear trend of the capacitance for smaller values of *G*, which becomes negligible with a larger *W*, in addition to a narrower dynamic range of the relative capacitance change Δ*C/C*_0_ that also corresponds to a lower sensitivity. The conditions *G = W =* 50 μm, and then, *Λ_m_* = 100 µm, have been chosen as the best compromise in terms of linearity and sensitivity, obtaining an impedance change up to 20% with a biofilm thickness of 15 μm. Since the detection of the changes of capacitance at the interface of the electrodes is not necessary, as required instead for the *INEs*, a frequency *f*_2_ of 1 kHz has been assumed for the simulation of the current (*i*_2_ in Figure 1a) changes with different thickness of the biofilm in order to probe electrical changes in the bulk solution [57].

**Figure 7:**
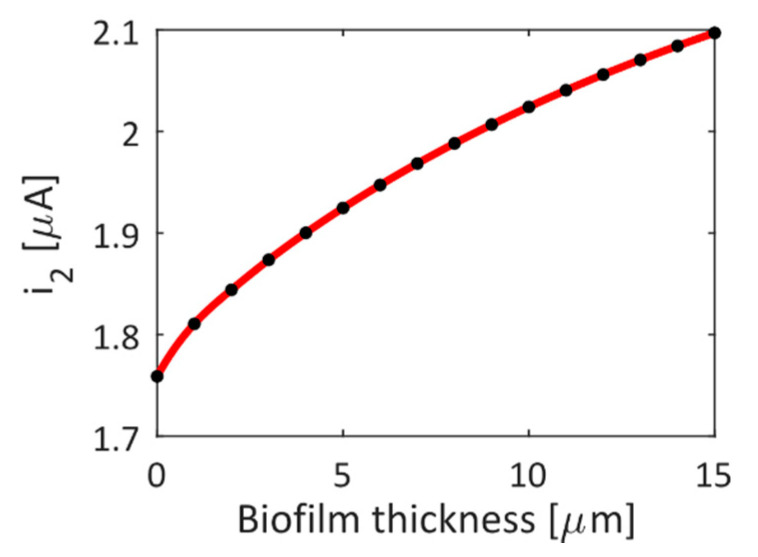
Current *i*_2_ [µA] in the *IME* structure with *N* = 100, *f*_2_ = 1 kHz, and *G* = *W* = 50 μm for different values of biofilm thickness. Plots calculated by 2D FEM approach.

The electrical performance by varying the biofilm thickness is reported in Figure 7. A change of the current *i*_2_ from *i*_2_ = 1.76 up to 2.09 μA with a quasi-linear behavior has been calculated, enabling the detection of the current change for each layer of biofilm that confirms the ability to easily detect both the bacterial biofilm growth and maturation, together with its possible disruption caused by the action of the antibiotics. A similar behavior represents a significant improvement for *AMR* because an efficient, accurate, and real-time analysis of the bacteria interaction and useful information about their life in community and colonies can be achieved. Furthermore, the electrical measurements of the *IMEs* ensure an accurate analysis of the metabolic state of the biofilm during the whole process from the formation to the maturation and, possibly, the disruption for specific antibiotics.

## 5. Discussions and Conclusions

An innovative label-free biosensing platform based on a dual array of interdigitated electrodes for simultaneous optical and electrical measurements has been proposed for the analysis of bacteria and their interaction. A multiparametric approach for the *INEs* section offers high sensitivity to detect a very low concentration of planktonic bacteria and possibly down to a single cell, also ensuring the following of the biofilm formation in the initial stage. In addition, the electrical performance of the *IMEs* enables the monitoring of the growth and the maturation of the biofilm to possibly investigate the efficiency of the antibiotics. The overall performance, related to the merging of the micro and nano scale, outperforms the competitive technologies, whose operation is limited to monitoring a few biofilm formation stages. Therefore, the main advantage of the proposed system is the capability to detect and monitor in real time a biofilm, also analyzing its metabolic state and evolution phase. In its proof-of-concept form, the proposed optoelectronic device has been used for the monitoring of *Escherichia coli*-based biofilm, but the device could also be investigated to analyze biofilms formed by different strains and species of bacteria. Therefore, the proposed detection method’s results are very promising due to high sensitivity, low-cost fabrication, and real-time operation, paving the way to a real-time and cost-effective solution to counteract the AMR phenomenon.

## Figures and Tables

**Table 1:** Electrical properties of the parameters used in the *MMT* [46,56].

	Conductivity [S/m]	Relative Permittivity [a.u.]
Cytoplasm (*CYT*)	0.220	100
Membrane (*MEM*)	10^−7^	10.8
Extracellular Matrix *(ECM)*	0.680	60
Lysogeny Broth *(LB)*	0.754	60

## Data Availability

Not applicable.
